# Liver Disease and Prevalence of Liver Transplantation in Adults With ZZ Alpha‐1 Antitrypsin Deficiency—A Meta‐Analysis

**DOI:** 10.1002/lci2.70013

**Published:** 2025-04-11

**Authors:** Adam M. Syanda, Dimitra Georgantaki, Muhammad Awsaf, Mariam Molokhia, S. Tamir Rashid

**Affiliations:** ^1^ Department of Metabolism, Digestion and Reproduction, Faculty of Medicine Imperial College London London UK; ^2^ Department of Population Health Sciences, School of Life Course and Population Sciences King's College London London UK

**Keywords:** alpha‐1 antitrypsin deficiency, cirrhosis, fibrosis, liver cancer, liver transplantation, PIZ, steatosis

## Abstract

Alpha‐1 antitrypsin deficiency (A1ATD) is an inherited metabolic disorder caused by a mutation (ZZ) in the SERPINA1 gene. Carriers are predisposed to liver and lung pathology. The severity of A1ATD‐associated liver disease is highly variable, necessitating further characterisation. This study aims to investigate the risk and extent of liver disease and the prevalence of liver transplantation in ZZ A1ATD patients. Several established databases, including Ovid, EBSCO, PubMed, and Cochrane Library, were searched from inception to May 12, 2024. Data were pooled using a random effects model, and study weight was calculated using the inverse variance method. Crude odds ratios (cOR) were calculated using participants with the MM genotype as the comparator. The study was registered in PROSPERO (CRD42022335666). Of the 4420 studies identified, 45 studies and 8638 A1ATD patients (38.8% female) were included. ZZ A1ATD patients demonstrate an increased risk of liver diseases compared to controls, including steatosis (crude odds ratio (cOR): 1.52 [95% CI: 1.21, 1.91]), fibrosis (cOR: 9.85 [95% CI: 5.70, 17.03]), cirrhosis (cOR: 10.43 [95% CI: 5.51, 19.73]), and liver cancers (cOR: 14.12 [95% CI: 6.50, 30.66]). The prevalence of liver transplantation is considerable, with rates reaching 5% [95% CI: 0.00, 12.34]. Our findings confirm the substantial burden of liver disease in ZZ A1ATD patients, including subclinical manifestations such as steatosis and fibrosis that may remain undetected. Given the lack of approved treatments for A1ATD‐associated liver disease, prioritising the development of novel therapies to stop or reverse liver disease is essential.

**Summary**

- ZZ A1ATD patients are at an increased risk of liver disease and liver cancer.
- One fifth of ZZ A1ATD patients experience transaminitis and fibrosis.
- ZZ A1ATD patients are at a 10‐times increased risk of developing cirrhosis.
- 5% of ZZ A1ATD patients required liver transplantation.

**Abbreviations**
A1ATD: alpha‐1 antitrypsin deficiency
ALP: alkaline phosphatase
ALT: alanine aminotransferase
aOR: adjusted odds ratio
AST: aspartate aminotransferase
CAP: controlled attenuation parameter
CI: confidence interval
cOR: crude odds ratio
GGT: gamma‐glutamyl transferase
HCC: hepatocellular carcinoma
LSM: liver stiffness measurement
MM: wildtype
SMD: standard mean difference

## 1 Introduction

Alpha‐1 antitrypsin deficiency (A1ATD) is a multisystemic inherited disorder affecting liver and lung function [1, 2, 3, 4]. A point mutation (c.1096G>A) in the SERPINA1 gene, which encodes the alpha‐1 antitrypsin protein (A1AT), results in the generation of the pathogenic Z allele (Glu366Lys) [5, 6, 7]. This variant results in the misfolding of A1AT, causing intracellular polymerisation in hepatocytes and failure of release into the bloodstream [6, 8]. The accumulation of these polymers has been associated with hepatocellular dysfunction [9], inflammation [10], cirrhosis [11], and hepatocellular carcinoma [10]. Additionally, the systemic deficiency has been linked with the progressive deterioration of lung function [12].

Currently, the most accurate prevalence estimates of A1ATD are derived from unbiased population‐based screening initiatives. For instance, a nation‐wide screening of 200 000 neonates in Sweden identified the frequency of the homozygous ZZ genotype as 1 in 1639 [13], while a similar screening of 107 038 in Oregon, US reported a frequency of 1 in 4455 [14]. Due to the geographic origins of the A1ATD ZZ mutation, this variant is predominantly observed in individuals of northern European descent [15]. It is estimated that approximately 200 000 people worldwide are homozygous carriers of the Z allele [16].

The clinical penetrance of A1ATD exhibits significant variability, with a bimodal age distribution for the onset of symptoms [17]. Patients typically present either in early childhood with liver pathology or late adulthood with liver or lung pathology [18]. In contrast to A1ATD‐related lung disease, where cigarette smoking is a major risk factor for disease progression [19], no equivalent strong risk factor has been identified for liver disease. Although environmental factors such as diet and alcohol intake have been suggested to interact with genetic factors and influence disease presentation, more research is necessary to clarify these associations, highlighting the need for comprehensive research to understand the underlying mechanisms and improve management strategies.

Despite the growing body of research on A1ATD, there is a critical need to consolidate data on the clinical outcomes associated with this condition. This systematic review and meta‐analysis aims to estimate the risk and extent of liver disease and the prevalence of liver transplantation in ZZ A1ATD patients.

## 2 Methods

### 2.1 Search Strategy and Selection Criteria

The search strategy employed a broad approach, ensuring the inclusion of all potentially relevant studies. Search terms are detailed in Figure S1. We identified A1ATD studies with explicit diagnoses of the ZZ genotype or PiZ phenotype from a comprehensive search of multiple databases. These included Ovid databases (MEDLINE, MEDLINE In Process, EMBASE, Global Health, Journals@Ovid), EBSCO databases (CINAHL), PubMed Central, Cochrane Library (Cochrane Reviews, Cochrane Protocols, Cochrane Central Register of Controlled Trials), EU Clinical Trials Register, NIHR Health Technology Assessment, NHS Economic Evaluation Database, Database of Abstracts of Reviews of Effects, Web of Science (Clarivate), ClinicalTrials.gov, ISRCTN Registry, WHO International Clinical Trials Registry Platform, and MedRxiv (preprints). All records from database inception to the search date (May 12, 2024) were included.

Exclusion criteria encompassed animal or cell‐based studies, publications solely investigating heterozygous A1ATD patients or genotypes other than ZZ, non‐English language publications, and case series studies. Paediatric or mixed‐age cohorts were also excluded. After manual and semi‐automated record deduplication, publications were screened by title and abstract by two independent reviewers in an online tool Rayyan [20], yielding 45 studies included in the review and reported as per PRISMA guidelines (Figure S2).

### 2.2 Data Analysis

Various study types were collated, including case series with more than five ZZ participants, cross‐sectional studies, case–control studies, cohort studies, and randomised controlled trials. Several studies lacked comparators or used heterozygous A1ATD variants as controls. For the purposes of crude odds ratio and standardised mean difference (SMD) analyses, only studies with MM (wildtype) comparators were selected. The preliminary study protocol was registered in PROSPERO (CRD42022335666).

Two independent reviewers extracted data from screened articles. The dataset included rates of steatosis, defined as controlled attenuation parameter (CAP) > 248 dB/m or Brunt's steatosis grade ≥ 1 or Kleiner score ≥ 1, fibrosis, defined as liver stiffness measurement (LSM) ≥ 7.1 kPa or Ishak score ≥ 2 or METAVIR score ≥ 2, cirrhosis, hepatocellular carcinoma (HCC) determined via registry entry or histological analysis, elevated liver enzymes, and liver transplantation rates. Discrepancies were resolved through discussions with senior consultants on the review team. The studies selected for odds ratio analyses had age‐ and gender‐matched controls. To avoid duplication, studies that shared patient cohorts were carefully reviewed, with priority given to newer or larger studies.

The risk of bias was assessed using the Newcastle‐Ottawa Scale, with specific criteria detailed in Figure S5, covering biases in selection, comparability, and outcome. Each study was evaluated by two independent reviewers and assigned a score, with rankings categorised as low, moderate, or high risk of bias. Studies that scored below a threshold value of 2 on the Newcastle–Ottawa Scale were excluded from the analysis.

Analyses were undertaken using Python packages pymeta and statsmodels. Prevalence was pooled using a random‐effects model with weighted least squares regression, and study weight was calculated using the inverse variance method. The proportions of outcomes were compared between ZZ cohorts and MM controls by recording case counts and performing crude odds ratio (cOR) analysis. Data confidence was reported as a 95% confidence interval (CI). This comparison was performed using inverse variance algorithms within a random effects model framework to account for variability across studies. For continuous data types, effect sizes were measured using standardised mean differences. The inverse variance Hedges–adjusted algorithm was applied, also under a random effects model, to provide a more robust estimation of effect sizes across diverse study designs.

Given the limited number of studies available for meta‐analysis, we examined two distinct groups: non‐hepatic recruitment group (Group 1), consisting of studies that did not specifically recruit patients based on liver disease, such as screening studies and those focused on the non‐hepatic indications associated with A1ATD, and hepatic recruitment group (Group 2), consisting of studies that specifically recruited patients due to the presence of liver disease. To assess the potential bias introduced by the hepatic recruitment group studies, both groups were analysed separately as subgroups.

Heterogeneity was assessed using Cochran's Q value, *I* ^2^ statistic, and the *τ* ^2^ statistics. Publication bias was evaluated by funnel plots of study size against the log‐odds of prevalence (Figure S6). All procedures adhered to PRISMA guidelines.

## 3 Results

Out of an initial 6899 studies identified, 4420 were screened after the removal of duplicates, and 45 met the eligibility criteria for inclusion in this meta‐analysis (Table S1, Figure S2). Studies were geographically distributed across Europe, North America, and South America, with the majority originating from the USA (*n* = 16), Sweden (*n* = 8) and the UK (*n* = 5) (Figure S3A,B). Of the included studies, 53.3% of selected studies (*n* = 24) were published in the last decade (Figure S3C). Cumulatively, there were 8638 participants in the ZZ cohorts and 685 992 participants in the comparator cohorts (MM genotype). Among the 45 eligible studies, 41 (91.1%) did not pre‐select participants based on pre‐existing liver pathology (Group 1), while 4 studies (8.9%) specifically included participants with known liver conditions (Group 2). These subsets were analysed separately to account for baseline differences in liver disease status. In Group 1, the mean participant age was 49.5 years [SD: 8.2], with female participants comprising 39.2% of the ZZ cohorts. Conversely, in Group 2, the mean age was 49.5 years [SD: 5.8], with females representing 31.5% of the study population in the ZZ cohorts. The control cohorts were aged 50.2 [SD: 11.4] and 51.8 [SD: 8.3] for Groups 1 and 2, respectively (Figure S4).

### 3.1 Clinical Manifestations of Liver Disease

**FIGURE 1:**
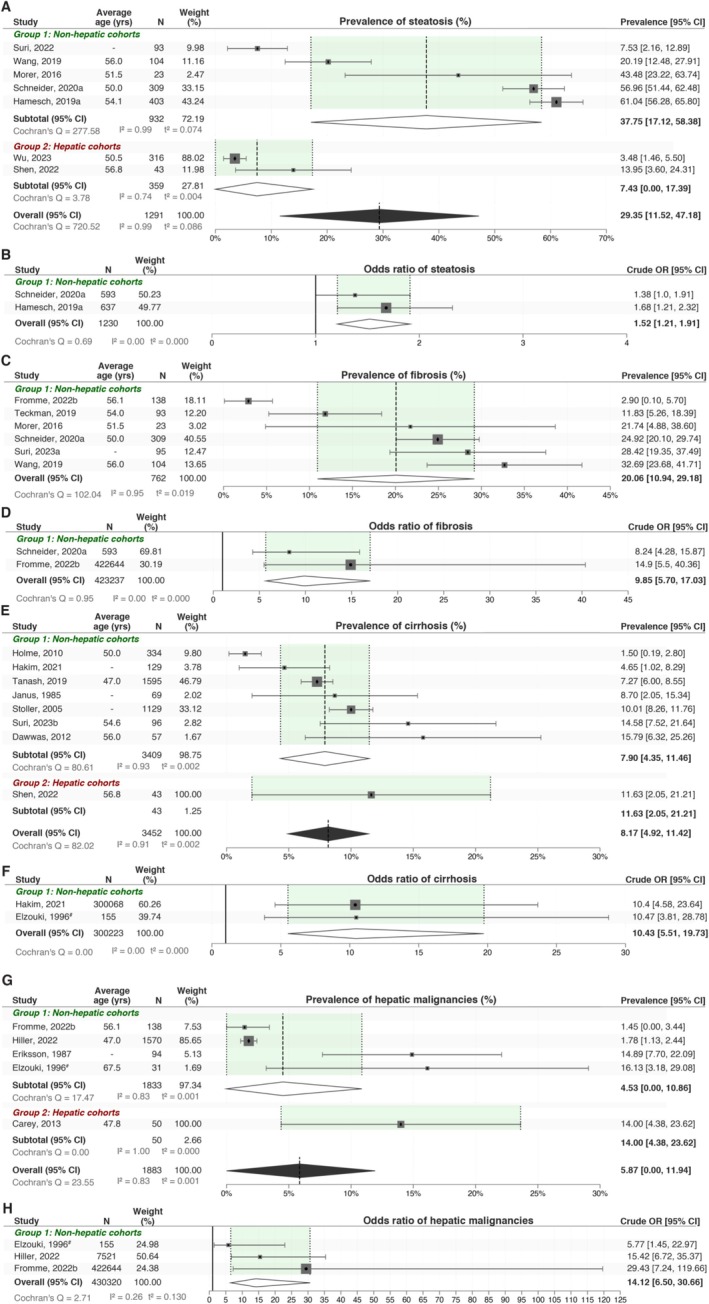
Elevated prevalence and crude odds ratios of hepatic conditions in ZZ A1ATD patients. Prevalence (A) and crude odds ratio (B) of steatosis demonstrate greater risk in ZZ A1ATD patients. Similarly, prevalence (C) and crude odds ratio (D) of fibrosis highlights increased risk with the ZZ genotype. The analysis for crude odds ratios was matched for age and gender between case and control groups. Prevalence (E) and crude odds ratio (F) of cirrhosis demonstrate greater risk in ZZ A1ATD patients. Similarly, prevalence and crude odds ratio of HCC are increased in the ZZ cohorts (G, H). The analysis for crude odds ratios was matched for age and gender between case and control groups. # = case–control study design.

In this study, we first quantified clinical manifestations of liver disease in ZZ A1ATD patients. Steatosis, often an early indicator of liver dysfunction [21, 22, 23] can present mildly but signals metabolic alterations [24]. The pooled prevalence of steatosis was 29.35% [95% CI: 11.52, 47.18], derived from 7 studies encompassing 1291 patients (Figure 1A). Crude odds ratio analysis revealed a pooled cOR of 1.52 [95% CI: 1.21, 1.91], underscoring a significantly higher incidence of steatosis in the ZZ cohort compared to MM controls (Figure 1B). Fibrosis, a hallmark of chronic liver injury and a precursor to more severe liver disease [23, 25] demonstrated a pooled prevalence of 20.06% [95% CI: 10.94, 29.18] (Figure 1C). The cOR for fibrosis was markedly elevated in ZZ patients compared to MM controls, with a pooled cOR of 9.85 [95% CI: 5.70, 17.03] (Figure 1D), indicating a substantially increased risk of fibrotic progression in the ZZ population.

Cirrhosis, a hallmark of advanced chronic liver disease [26], demonstrated an overall pooled prevalence of 8.17% [95% CI: 4.92, 11.42] (Figure 1E). This analysis included data from seven studies that did not specifically recruit patients for liver disease (non‐hepatic recruitment group), encompassing a total of 3409 patients, which reported a cirrhosis prevalence of 7.90% [95% CI: 4.35, 11.46]. In contrast, one study (*N* = 43; 1.25% total weight) that specifically recruited participants with liver disease (hepatic recruitment group) reported a significantly higher cirrhosis prevalence of 11.63% [95% CI: 2.05, 21.21]. The cOR for cirrhosis in controlled studies comparing ZZ patients to the MM population was notably elevated at 10.43 [95% CI: 5.51, 19.73] (Figure 1F). The development of a liver malignancy, such as hepatocellular carcinoma, is often indicative of long‐term injury and inflammation [10]. The pooled prevalence of liver cancers across five studies was 5.87% [95% CI: 0.00, 11.94]. This included data from four studies not selecting for liver disease (non‐hepatic recruitment group), involving 1833 participants, which reported an HCC prevalence of 4.53% [95% CI: 0.00, 10.86] (Figure 1G). In contrast, one study specifically recruiting for liver disease (hepatic recruitment group), with a total of 50 participants, revealed a markedly higher HCC prevalence of 14.00% [95% CI: 4.38, 23.62]. The cOR for HCC was 14.12 [95% CI: 6.50, 30.66] (Figure 1H), highlighting the increased risk of liver cancer in the ZZ cohort.

### 3.2 Blood Markers

**FIGURE 2:**
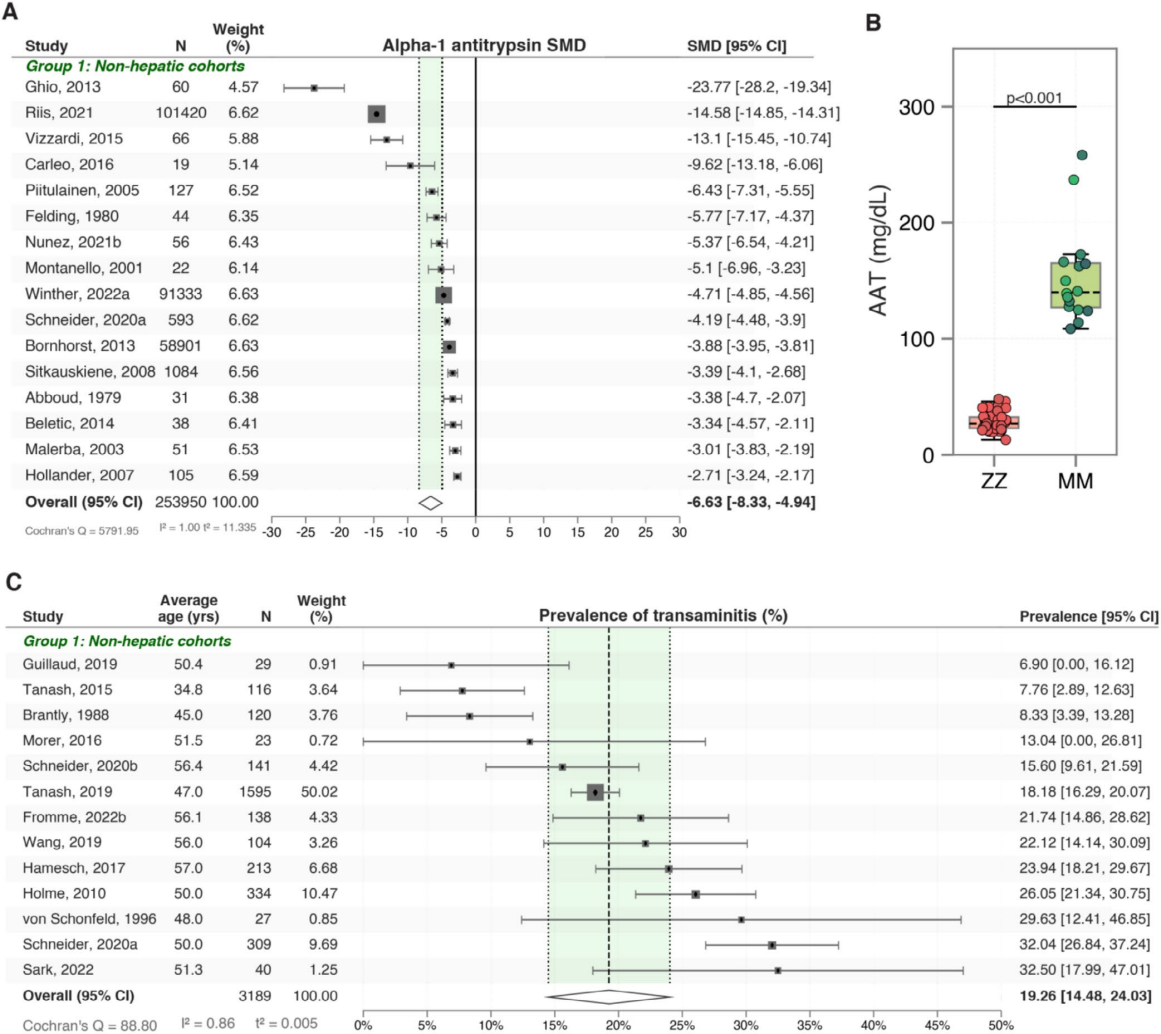
Severe serum alpha‐1 antitrypsin deficiency and high prevalence of transaminitis in ZZ A1ATD patients. The shortlisted studies consistently demonstrate a deficiency of A1AT in every cohort of ZZ patients (A, B). Each datapoint on the boxplot signifies a mean cohort AAT level from a single study. The differences between means were assessed by a *t*‐test. Elevated liver enzymes are a frequent finding in ZZ A1ATD cohorts (C).

Although serum alpha‐1 antitrypsin levels are not directly indicative of liver injury, we considered their levels to determine the magnitude of deficiency in ZZ cohorts. Our analysis, derived from 16 studies involving 253 950 participants, revealed a pooled SMD for serum AAT levels of −6.63 [95% CI: −8.33, −4.94] (Figure 2A). This significant deficiency was further highlighted by the comparison of absolute serum levels between MM and ZZ patients, where MM patients exhibited notably higher mean serum AAT levels at 164.83 mg/dL [SD: 61.09] compared to ZZ patients at 29.06 mg/dL [SD: 8.57] (*p* < 0.001) (Figure 2B).

Serum biomarkers provide a valuable non‐invasive means of assessing liver injury. We focused on liver enzyme levels as the most commonly used indicators. We found that in 13 studies, a pooled prevalence of elevated liver enzymes (transaminitis) was 19.26% [95% CI: 14.48, 24.03] (Figure 2C) in 3189 participants.

**FIGURE 3:**
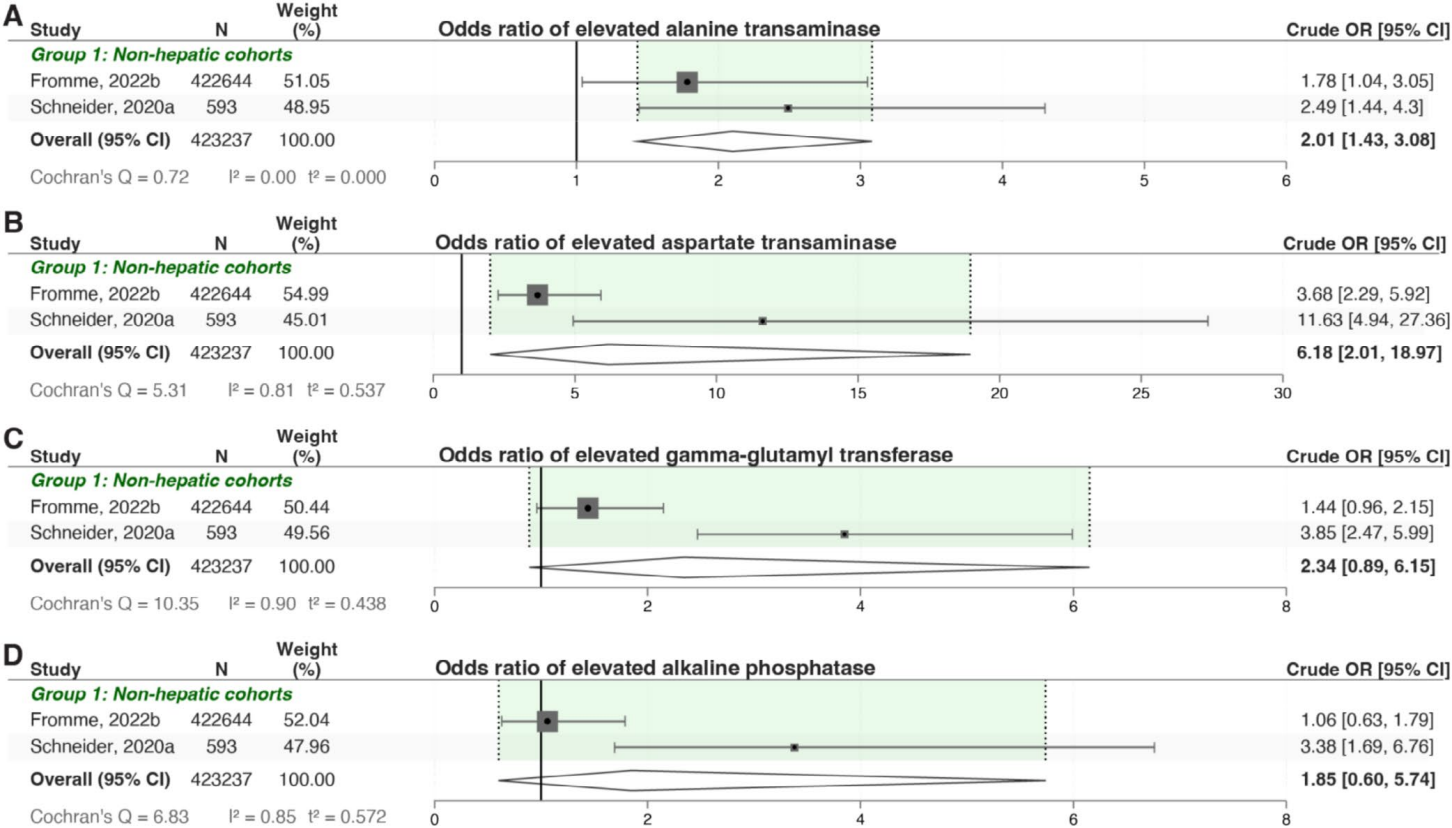
ZZ A1ATD patients are at an increased risk of elevated liver enzymes. Odds ratio analysis (A–D) demonstrate that alanine transaminase (ALT) and aspartate transaminase (AST) were significantly elevated in ZZ patients compared to MM controls, and gamma‐glutamyl transferase (GGT) and alkaline phosphatase (ALP) were marginally elevated in ZZ patients compared to MM controls. The analysis for crude odds ratios was matched for age and gender between case and control groups.

We then investigated derangement of individual liver enzymes, including alanine aminotransferase (ALT), aspartate aminotransferase (AST), gamma‐glutamyl transferase (GGT), and alkaline phosphatase (ALP) (Figure 3). The comparative analysis was based on controlled shortlisted studies for each marker. The results indicated elevated cORs across all markers, with significantly elevated ALT showing a cOR of 2.10 [95% CI: 1.43, 3.08], significantly raised AST at a cOR 6.18 [95% CI: 2.01, 18.97], GGT at a cOR 2.34 [95% CI: 0.89, 6.15], and ALP at a cOR 1.85 [95% CI: 0.6, 5.74] (Figure 3A–D).

### 3.3 Liver Transplantation

**FIGURE 4:**
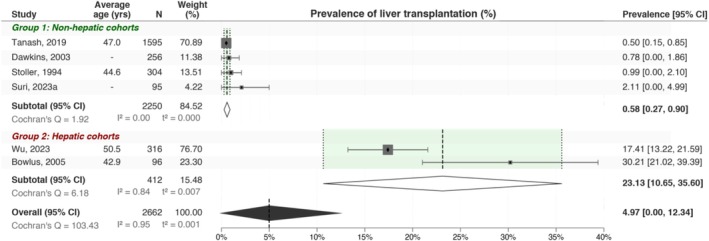
Liver transplantation prevalence in A1ATD ZZ patients. The prevalence of liver transplantation is highly heterogeneous between two cohort subgroups.

Chronic liver injury in A1ATD can culminate in liver failure [27] necessitating liver transplantation. Consequently, we investigated the rates of liver transplantation and estimated the pooled prevalence of 4.97% [95% CI: 0.00, 12.34] (Figure 4). The substantial heterogeneity observed in these rates is likely attributable to variations in sample sizes and recruitment biases, particularly between the non‐hepatic recruitment group and the hepatic recruitment group.

## 4 Conclusion

This systematic review aimed to provide a comprehensive meta‐analysis of current literature on A1ATD‐related liver disease, encompassing data from 8638 participants across 45 studies. By analysing the prevalence of clinical and biochemical evidence of liver pathology, this review seeks to provide insights into the disease penetrance and risks of transplantation in ZZ A1ATD cohorts. We conducted analyses in two subgroups based on the recruitment criteria of the participants. Some studies employed relatively unbiased recruitment methods, such as screening initiatives, while others specifically selected patients based on the presence of conditions unrelated to liver disease, such as lung pathology [28], panniculitis [29], or thromboembolism [30] (non‐hepatic recruitment group). Conversely, other studies specifically recruited patients with liver conditions, such as fibrosis or cirrhosis (hepatic recruitment group), thereby introducing a selection bias.

Approximately one‐third of ZZ A1ATD patients (29.35% [95% CI: 11.52, 47.18]) exhibited steatosis, with a cOR of 1.52 [95% CI: 1.21, 1.91]. The increased rates of steatosis may suggest a potential predisposition to MASLD, which is consistent with the “two‐hit” hypothesis of liver injury in A1ATD [31]. The first “hit” stems from the accumulation of misfolded alpha‐1 antitrypsin proteins in the liver, influenced by genetic factors, leading to hepatocellular stress and damage. The second “hit” arises from additional metabolic insults, such as obesity, insulin resistance, or alcohol consumption, which exacerbate the hepatic injury and promote the progression to more severe conditions at a higher rate than in the setting of injury in MM controls.

Our findings further demonstrate a notable prevalence of fibrosis (20.06% [95% CI: 10.94, 29.18]) with a cOR of 9.85 [95% CI: 5.70, 17.03], indicating almost a tenfold increased risk in the ZZ cohort. Similarly, cirrhosis was present in 8.17% [95% CI: 4.92, 11.42] of patients, with a cOR of 10.43 [95% CI: 5.51, 19.73], in line with previous reports by Townsend et al. (2018) (10%) [32]. The pooled prevalence of liver cancer was 5.87% [95% CI: 0.00, 11.94], with a cOR of 14.12 [95% CI: 6.50, 30.66], suggesting a significantly increased risk of malignancy in ZZ A1ATD patients.

Other studies such as Fromme et al. (2022) which investigate the prevalence of liver disease in ZZ patients within the UK Biobank, report a higher risk of fibrosis/cirrhosis (adjusted OR (aOR) 21.7 [95% CI: 8.8, 53.7]) and liver cancer (aOR 44.5 [95% CI: 10.8183.6]) from ZZ A1ATD patients in the UK Biobank compared to our meta‐analysis. Their studies incorporate patient‐level data such as age, sex, body mass index, diabetes status, and alcohol consumption in the calculation of adjusted odds ratios—a level of detail unachievable in the meta‐analysis due to the nature of aggregate data from the shortlisted studies. Despite these marginal methodological differences, the discrepancy between their results and ours accentuates the findings of our meta‐analysis—a substantial predisposition to severe liver disease within the ZZ A1ATD cohort.

While early detection of liver complications in A1ATD may not yet facilitate the application of curative treatment options, ongoing clinical trials are exploring potential therapies designed to prevent or reverse liver damage in these patients [33, 34]. Monitoring strategies for liver complications remain important, as they can help stratify patients who may benefit from emerging treatments. Additionally, lifestyle modifications, including dietary management and reducing alcohol intake, are essential in mitigating additional “hits” that contribute to liver pathology in A1ATD.

There is a critical need for non‐invasive methods to identify ZZ A1ATD patients at risk for liver disease progression, particularly for enrolment in clinical trials targeting emerging therapies. Non‐invasive biomarkers can help stratify patients and monitor disease progression, offering a practical alternative to liver biopsies. Our analysis revealed that approximately one‐fifth (19.26% [95% CI: 14.48, 24.03]) of ZZ A1ATD patients exhibit elevated liver enzyme levels, suggestive of liver injury. Specifically, significant elevations were observed in ALT (cOR 2.10 [95% CI: 1.43, 3.08]), AST (cOR 6.18 [95% CI: 2.01, 18.97]), GGT (cOR 2.34 [95% CI: 0.89, 6.15]), and ALP (cOR 1.85 [95% CI: 0.60, 5.74]). Although liver enzyme levels have been criticised for their lack of specificity in predicting liver injury in A1ATD [35], they can still provide valuable insights. Elevated liver enzymes indicate hepatic injury, which, when combined with other non‐invasive measures, could improve the identification of patients at risk for more severe liver conditions.

Finally, the prevalence of liver transplants showed significant variation between the groups, with 0.58% (95% CI: 0.27, 0.90) observed in the non‐hepatic recruitment group compared to 23.13% (95% CI: 10.65, 35.60) in the hepatic recruitment group. The difference between subgroups is due to the selection bias inherent in the hepatic recruitment group. This is substantially higher than the transplantation rates in the general population in the United Kingdom (0.02%) [36], highlighting the increased risk of severe liver disease progression necessitating a liver transplant in A1ATD ZZ patients with and without pre‐existing liver disease.

These data highlight the importance of monitoring liver function in all adults diagnosed with AATD as well as promoting patient education, which emphasises preventative and protective strategies regardless of current liver function. Additionally, screening programmes may be of benefit in areas of high prevalence by assisting in the identification of people with subclinical AATD, who may then be targeted with data‐driven preventative advice. The increased awareness of liver complications may lead to lifestyle changes, such as reduced alcohol intake and thus prevention of accelerated disease progression.

## 5 Strengths and Limitations

Our analysis draws on data from over 8600 participants across 45 studies conducted in more than 12 countries, providing a broad and diverse dataset. The studies are representative of the population most at risk, as A1ATD is primarily found in individuals of Northern European ethnicity. The inclusion of studies beyond the endemic region enhances the generalisability of our findings.

Several limitations must be acknowledged. High study heterogeneity limited the number of publications included. Confounders, such as comorbidities, were not accounted for due to the absence of disaggregated data. Many studies were excluded for lacking genotype‐specific data, and some were included only for selected parameters due to missing data on other outcomes, restricting the inclusion of all studies for all outcomes described in our meta‐analysis. The studies included in the meta‐analysis also exhibited some heterogeneity, as is expected with case series and observational cohort studies. Most studies included in this review were retrospective and recruited patients from clinical settings or existing registries. This recruitment strategy likely biases the data towards individuals who sought medical attention, potentially under‐representing those with the ZZ variant with less severe symptoms [37]. As a result, our findings may not fully capture the liver disease burden in the broader A1ATD population. This further underscores the importance of prospective designs that begin with asymptomatic or minimally symptomatic A1ATD ZZ patients, enabling the capture of even earlier hepatic changes and refining our understanding of disease progression. Moreover, incorporating non‐invasive tools, such as transient elastography and novel biomarkers, may provide robust data on progression rates, potential risk factors, and the timing of clinical interventions.

While the ZZ homozygous patients have been reported to suffer the most severe manifestations of A1ATD, it is crucial to investigate other heterozygous carriers with higher overall prevalence to understand the full spectrum of adult‐onset liver disease. of particular clinical interest is the MZ genotype as patients are known to be at an increased risk of liver disease [38, 39] and the estimated global population surpasses 35 million [40]. In addition, exploring liver disease in children with ZZ A1ATD could provide valuable insights, as they often present with more severe pathology than adults.

Funnel plots used to assess publication bias displayed mostly symmetrical patterns, though a degree of asymmetry was observed (Figure S6). This asymmetry was not considered detrimental due to the low number of studies involved. Additionally, a bias assessment using the Newcastle‐Ottawa Scale on the included studies indicated potential sources of bias in the results. Specifically, 21 studies (42.9%) scored ≥ 6, suggesting a low risk of bias, while 22 studies (44.9%) scored between 4 and 5, indicating a moderate risk, and six studies (12.2%) scored ≤ 3, classified as high risk (Figure S5). While studies scoring 2 or below were removed from downstream analyses, extra care should be taken when interpreting the results from studies scored as high risk.

## Author Contributions

A.M.S. and M.A. conducted the research and data collection. A.M.S. and D.G. performed data analysis. A.M.S. designed the research study. The manuscript was written by A.M.S., D.G., and M.A., M.M. and S.T.R. provided critical review and revisions of the manuscript. All authors reviewed and approved the final version of the manuscript.

## Conflicts of Interest

The authors declare no conflicts of interest.

## Supporting information


Data S1.

**TABLE S1.** Search terms used in the systematic review.

## Data Availability

The data that support the findings of this study are available from the corresponding author upon reasonable request.
