# Supplementary material for: Liver Disease and Prevalence of Liver Transplantation in Adults With ZZ Alpha‐1 Antitrypsin Deficiency—A Meta‐Analysis
Source: Liver Int Commun. 2025 Apr 11;6(2):e70013. doi: 10.1002/lci2.70013 (PMC12001869; doi:10.1002/lci2.70013)
Supplement: Supplementary file 1 — Data S1. [file LCI2-6-e70013-s001.pdf]

Liver disease and prevalence of liver transplantation in adults with ZZ alpha-1 antitrypsin deficiency – a meta-analysis

Authors: Adam M. Syanda, Dimitra Georgantaki, Muhammad Awsaf, Mariam Molokhia, S. Tamir Rashid

Supplementary material

Supplementary Figure 1

OVID databases

- MEDLINE (Ovid)
- EMBASE Classic and EMBASE (Ovid)
- Global Health (Ovid)
- Journals@Ovid (Ovid)

Step	Search term
1	(a-atd or a1-atd or a1at or a1atd or aatd or anti-tryps$ or antitryps$ or one-anti-tryps$ or one-antitryps$ or anti-proteinas$ or antiproteinas$ or alpha-1-anti-proteinas$ or alpha-1-anti-tryps$ or alfa-1-anti-tryps$ or alfa-1-antitryps$ or alpha-1-antiproteinas$ or alpha-1-antitryps$ or alpha-1-at or alpha-1-atd or alpha-one-anti-tryps$ or alpha-one-antitryps$ or alpha1-anti-proteinas$ or alpha1-anti-tryps$ or alpha1-antiproteinas$ or alpha1-antitryps$ or alpha1-at or proteinase inhibito$ or alpha1-proteinase inhibito$ or alpha-1-proteinase inhibito$ or 1-proteinase inhibito$ or one-proteinase inhibito$ or alpha1-atd or piz or pizz).ti,ab.
2	1 and (defic$ or lack$).ti,ab.
3	2 not (exp animals/ not humans.sh.)
4	3 not (mouse or murine or mice or "in vitro" or model).ti,ab.
5	4 and (OLT or transplant$ or morb$ or comorb$ or morbidi$ or decompensat$ or fail$ or diseas$ or disord$ or patho$ or abnorm$ or indication$ or hepatit$ or hepato$ or carcinom$ or cance$ or risk$ or prevalen$ or inciden$ or predict$ or associate$).ti,ab.
6	5 not review.pt.
7	remove duplicates from 6

Cochrane Library: Reviews, Protocols, Clinical Trials

Step	Search term
#1	(a1at or a1atd or aatd or anti-tryps* or antitryps* or one-anti-tryps* or one-antitryps* or anti-proteinase or antiproteinase or piz or pizz or " zz "):ti,ab,kw
#2	(defic* OR lack*):ti,ab,kw
#3	#1 and #2
#4	(OLT or transplant* or morb* or comorb* or morbidi* or decompensat* or fail* or diseas* or disord* or patho* or abnorm* or indication* or hepatit* or hepato* or carcinom* or cance* or risk* or prevalen* or inciden* or predict* or associated):ti,ab,kw
#5	#3 and #4

EU Clinical Trials Register

**Search term**
anti-trypsin deficiency OR antitrypsin deficiency OR pizz

NHS EED/DARE/HTA

**Search term (Title)**
anti-trypsin OR antitrypsin

EBSCO database

- CINAHL (EBSCO)

**Step**	**Search term**	**Search Options**
S1	TI (a-atd or a1-atd or a1at or a1atd or aatd or anti-tryps* or antitryps* or one-anti-tryps* or one-antitryps* or anti-proteinase or antiproteinase or alpha-1-anti-proteinas* or alpha-1-anti-tryps* or alfa-1-anti-tryps* or alfa-1-antitryps* or alpha-1-antiproteinas* or alpha-1-antitrypsin or alpha-1-at or alpha-1-atd or alpha-one-anti-trypsin or alpha-one-antitrypsin or alpha1-anti-proteinase or alpha1-anti-trypsin or alpha1-antiproteinase or alpha1-antitryps* or alpha1-at or proteinase inhibito* or alpha1-proteinase inhibito* or alpha-1-proteinase inhibito* or 1-proteinase inhibito* or one-proteinase inhibito* or alpha1-atd or piz or pizz)	**Expanders** - Apply related words; Apply equivalent subjects<br>**Search modes** - Boolean/Phrase
S2	AB (a-atd or a1-atd or a1at or a1atd or aatd or anti-tryps* or antitryps* or one-anti-tryps* or one-antitryps* or anti-proteinase or antiproteinase or alpha-1-anti-proteinas* or alpha-1-anti-tryps* or alfa-1-anti-tryps* or alfa-1-antitryps* or alpha-1-antiproteinas* or alpha-1-antitrypsin or alpha-1-at or alpha-1-atd or alpha-one-anti-trypsin or alpha-one-antitrypsin or alpha1-anti-proteinase or alpha1-anti-trypsin or alpha1-antiproteinase or alpha1-antitryps* or alpha1-at or proteinase inhibito* or alpha1-proteinase inhibito* or alpha-1-proteinase inhibito* or 1-proteinase inhibito* or one-proteinase inhibito* or alpha1-atd or piz or pizz)	**Expanders** - Apply related words; Apply equivalent subjects<br>**Search modes** - Boolean/Phrase
S3	S1 or S2	**Expanders** - Apply equivalent subjects<br>**Search modes** - Boolean/Phrase
S4	TI (defic* or lack*)	**Expanders** - Apply related words; Apply equivalent subjects<br>**Search modes** - Boolean/Phrase
S5	AB (defic* or lack*)	**Expanders** - Apply related words; Apply equivalent subjects<br>**Search modes** - Boolean/Phrase
S6	S4 or S5	**Expanders** - Apply equivalent subjects<br>**Search modes** - Boolean/Phrase

S7	S3 and S6	**Expanders** - Apply equivalent subjects<br>**Search modes** - Boolean/Phrase
S8	TI (OLT or transplant* or morb* or comorb* or morbidi* or decompensat* or fail* or diseas* or disord* or patho* or abnorm* or indication* or hepatit* or hepato* or carcinom* or cance* or risk* or prevalen* or inciden* or predict* or associated)	**Expanders** - Apply related words; Apply equivalent subjects<br>**Search modes** - Boolean/Phrase
S9	AB (OLT or transplant* or morb* or comorb* or morbidi* or decompensat* or fail* or diseas* or disord* or patho* or abnorm* or indication* or hepatit* or hepato* or carcinom* or cance* or risk* or prevalen* or inciden* or predict* or associated)	**Expanders** - Apply related words; Apply equivalent subjects<br>**Search modes** - Boolean/Phrase
S10	S8 or S9	**Expanders** - Apply equivalent subjects<br>**Search modes** - Boolean/Phrase
S11	S7 and S10	**Expanders** - Apply equivalent subjects<br>**Search modes** - Boolean/Phrase
S12	S11 NOT (((MH "Animals+") OR (MH "Animal Studies") OR (TI "animal model*")) NOT (MH "human"))	**Expanders** - Apply equivalent subjects<br>**Search modes** - Boolean/Phrase
S13	S12 NOT (mouse or murine or mice or "in vitro" or model)	**Expanders** - Apply related words; Apply equivalent subjects<br>**Search modes** - Boolean/Phrase

PubMed Central

**Step**	**Search term**
#1	a-atd[Abstract] OR a1-atd[Abstract] OR a1at[Abstract] OR a1atd[Abstract] OR aatd[Abstract] OR anti-tryps*[Abstract] OR antitryps*[Abstract] OR one-anti-tryps*[Abstract] OR one-antitryps*[Abstract] OR anti-proteinase[Abstract] OR antiproteinase[Abstract] OR alpha-1-anti-proteinas*[Abstract] OR alpha-1-anti-tryps*[Abstract] OR alfa-1-anti-tryps*[Abstract] OR alfa-1-antitryps*[Abstract] OR alpha-1-antiproteinas*[Abstract] OR alpha-1-antitrypsin[Abstract] OR alpha-1-at[Abstract] OR alpha-1-atd[Abstract] OR alpha-one-anti-trypsin[Abstract] OR alpha-one-antitrypsin[Abstract] OR alpha1-anti-proteinase[Abstract] OR alpha1-anti-trypsin[Abstract] OR alpha1-antiproteinase[Abstract] OR alpha1-antitryps*[Abstract] OR alpha1-at[Abstract] OR proteinase inhibito*[Abstract] OR alpha1-proteinase inhibito*[Abstract] OR alpha-1-proteinase inhibito*[Abstract] OR 1-proteinase inhibito*[Abstract] OR proteinase inhibito*[Abstract] OR alpha1-atd[Abstract] OR piz[Abstract] OR pizz[Abstract]
#2	a-atd[Title] OR a1-atd[Title] OR a1at[Title] OR a1atd[Title] OR aatd[Title] OR anti-tryps*[Title] OR antitryps*[Title] OR one-anti-tryps*[Title] OR one-antitryps*[Title] OR anti-proteinase[Title] OR antiproteinase[Title] OR alpha-1-anti-proteinas*[Title] OR alpha-1-anti-tryps*[Title] OR alfa-1-anti-tryps*[Title] OR alfa-1-antitryps*[Title] OR alpha-1-antiproteinas*[Title] OR alpha-1-antitrypsin[Title] OR alpha-1-at[Title] OR alpha-1-atd[Title] OR alpha-one-anti-trypsin[Title] OR alpha-one-antitrypsin[Title] OR alpha1-anti-proteinase[Title] OR alpha1-anti-trypsin[Title] OR alpha1-antiproteinase[Title] OR alpha1-antitryps*[Title] OR alpha1-at[Title] OR proteinase inhibito*[Title] OR alpha1-proteinase inhibito*[Title] OR alpha-1-proteinase inhibito*[Title] OR 1-proteinase inhibito*[Title] OR one-proteinase inhibito*[Title] OR alpha1-atd[Title] OR piz[Title] OR pizz[Title]
#3	#1 or #2

#4	#3 AND ((defic*[Abstract] OR lack*[Abstract]) OR (defic*[Title] OR lack*[Title]))
#5	OLT[Abstract] OR transplant*[Abstract] OR morb*[Abstract] OR comorb*[Abstract] OR morbidi*[Abstract] OR decompensat*[Abstract] OR fail*[Abstract] OR disease[Abstract] OR diseases[Abstract] OR disord*[Abstract] OR pathol*[Abstract] OR abnorm*[Abstract] OR indication*[Abstract] OR hepatit*[Abstract] OR hepatoc*[Abstract] OR carcinom*[Abstract] OR cancer*[Abstract] OR risk*[Abstract] OR prevalen*[Abstract] OR inciden*[Abstract] OR predict*[Abstract] OR associated[Abstract]
#6	OLT[Title] OR transplant*[Title] OR morb*[Title] OR comorb*[Title] OR morbidi*[Title] OR decompensat*[Title] OR fail*[Title] OR disease[Title] OR diseases[Title] OR disord*[Title] OR pathol*[Title] OR abnorm*[Title] OR indication*[Title] OR hepatit*[Title] OR hepatoc*[Title] OR carcinom*[Title] OR cancer*[Title] OR risk*[Title] OR prevalen*[Title] OR inciden*[Title] OR predict*[Title] OR associated[Title]
#7	#5 OR #6
#8	#4 AND #7
#9	#8 NOT (animals [mh] NOT humans [mh])
#10	mouse[Abstract] OR mice[Abstract] OR murine[Abstract] OR “in vitro”[Abstract] OR model[Abstract]
#11	mouse[Title] OR mice[Title] OR murine[Title] OR “in vitro”[Title] OR model[Title]
#12	#10 OR #11
#13	#9 NOT #12
#14	#13 NOT "is retracted"[filter]

PubMed

Step	Search term
#1	a-atd[Title/Abstract] OR a1-atd[Title/Abstract] OR a1at[Title/Abstract] OR a1atd[Title/Abstract] OR aatd[Title/Abstract] OR anti-tryps*[Title/Abstract] OR antitryps*[Title/Abstract] OR one-anti-tryps*[Title/Abstract] OR one-antitryps*[Title/Abstract] OR anti-proteinase[Title/Abstract] OR antiproteinase[Title/Abstract] OR alpha-1-anti-proteinas*[Title/Abstract] OR alpha-1-anti-tryps*[Title/Abstract] OR alfa-1-anti-tryps*[Title/Abstract] OR alfa-1-antitryps*[Title/Abstract] OR alpha-1-antiproteinas*[Title/Abstract] OR alpha-1-antitrypsin[Title/Abstract] OR alpha-1-at[Title/Abstract] OR alpha-1-atd[Title/Abstract] OR alpha-one-anti-trypsin[Title/Abstract] OR alpha-one-antitrypsin[Title/Abstract] OR alpha1-anti-proteinase[Title/Abstract] OR alpha1-anti-trypsin[Title/Abstract] OR alpha1-antiproteinase[Title/Abstract] OR alpha1-antitryps*[Title/Abstract] OR alpha1-at[Title/Abstract] OR proteinase inhibito*[Title/Abstract] OR alpha1-proteinase inhibito*[Title/Abstract] OR alpha-1-proteinase inhibito*[Title/Abstract] OR 1-proteinase inhibito*[Title/Abstract] OR proteinase inhibito*[Title/Abstract] OR alpha1-atd[Title/Abstract] OR piz[Title/Abstract] OR pizz[Title/Abstract]
#2	#1 AND (defic*[Title/Abstract] OR lack*[Title/Abstract])
#3	#2 AND (OLT[Title/Abstract] OR transplant*[Title/Abstract] OR morb*[Title/Abstract] OR comorb*[Title/Abstract] OR morbidi*[Title/Abstract] OR decompensat*[Title/Abstract] OR fail*[Title/Abstract] OR disease[Title/Abstract] OR diseases[Title/Abstract] OR disord*[Title/Abstract] OR pathol*[Title/Abstract] OR abnorm*[Title/Abstract] OR indication*[Title/Abstract] OR hepatit*[Title/Abstract] OR hepatoc*[Title/Abstract] OR carcinom*[Title/Abstract] OR cancer*[Title/Abstract] OR risk*[Title/Abstract] OR prevalen*[Title/Abstract] OR inciden*[Title/Abstract] OR predict*[Title/Abstract] OR associated[Title/Abstract])

#4	#3 NOT (animals [mh] NOT humans [mh])
#5	#4 NOT (mouse[Title/Abstract] OR mice[Title/Abstract] OR murine[Title/Abstract] OR "in vitro"[Title/Abstract] OR model[Title/Abstract])
#6	#5 NOT (comment[pt] OR review[pt] OR book[pt] OR booksdocs[pt])

Clinical Trials.gov

Search term
antitrypsin OR anti-trypsin

ISRCNT Registry

Search term
antitrypsin OR anti-trypsin AND deficiency

WHO ICTRP

Search term
(antitrypsin deficiency) OR (anti-trypsin deficiency)

MedRxiv

Search term
antitrypsin deficiency

Web of Science (Clarivate)

Step	Search term
#1	TI=(a-atd or a1-atd or a1at or a1atd or aatd or anti-tryps* or antitryps* or one-anti-tryps* or one-antitryps* or anti-proteinase or antiproteinase or alpha-1-anti-proteinas* or alpha-1-anti-tryps* or alfa-1-anti-tryps* or alfa-1-antitryps* or alpha-1-antiproteinas* or alpha-1-antitrypsin or alpha-1-at or alpha-1-atd or alpha-one-anti-trypsin or alpha-one-antitrypsin or alpha1-anti-proteinase or alpha1-anti-trypsin or alpha1-antiproteinase or alpha1-antitryps* or alpha1-at or proteinase inhibito* or alpha1-proteinase inhibito* or alpha-1-proteinase inhibito* or 1-proteinase inhibito* or one-proteinase inhibito* or alpha1-atd or piz or pizz)
#2	AB=(a-atd or a1-atd or a1at or a1atd or aatd or anti-tryps* or antitryps* or one-anti-tryps* or one-antitryps* or anti-proteinase or antiproteinase or alpha-1-anti-proteinas* or alpha-1-anti-tryps* or alfa-1-anti-tryps* or alfa-1-antitryps* or alpha-1-antiproteinas* or alpha-1-antitrypsin or alpha-1-at or alpha-1-atd or alpha-one-anti-trypsin or alpha-one-antitrypsin or alpha1-anti-proteinase or alpha1-anti-trypsin or alpha1-antiproteinase or alpha1-antitryps* or alpha1-at or proteinase inhibito* or alpha1-proteinase inhibito* or alpha-1-proteinase inhibito* or 1-proteinase inhibito* or one-proteinase inhibito* or alpha1-atd or piz or pizz)
#3	#1 OR #2

#4	TI=(defic* or lack*)
#5	AB=(defic* or lack*)
#6	#4 OR #5
#7	#3 AND #6
#8	TI=(OLT or transplant* or morb* or comorb* or morbidi* or decompensat* or fail* or diseas* or disord* or patho* or abnorm* or indication* or hepatit* or hepato* or carcinom* or cance* or risk* or prevalen* or inciden* or predict* or associated)
#9	AB=(OLT or transplant* or morb* or comorb* or morbidi* or decompensat* or fail* or diseas* or disord* or patho* or abnorm* or indication* or hepatit* or hepato* or carcinom* or cance* or risk* or prevalen* or inciden* or predict* or associated)
#10	#8 OR #9
#11	#7 AND #10
#12	TI=(mouse or murine or mice or "in vitro" or model)
#13	AB=(mouse or murine or mice or "in vitro" or model)
#14	#12 OR #13
#15	#11 NOT #14

Supplementary Figure 2

A

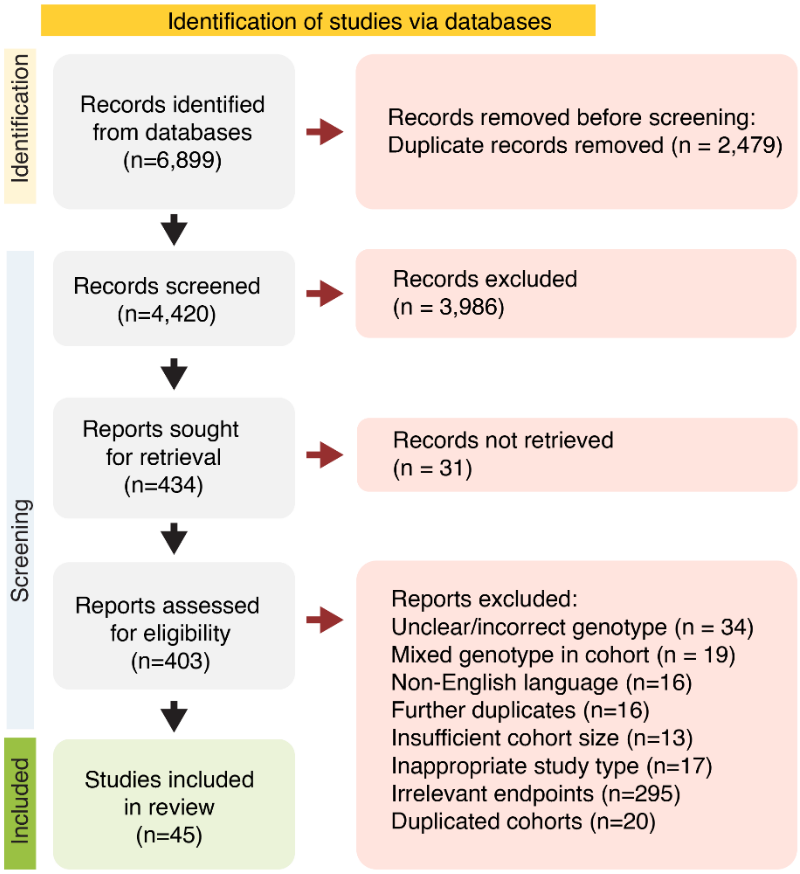

Supplementary Figure 2: PRISMA flowchart illustrating the study selection process. Of 6,899 records, 2,479 duplicates were removed. After screening and exclusions, 45 studies were included in the final review.

Supplementary Figure 3

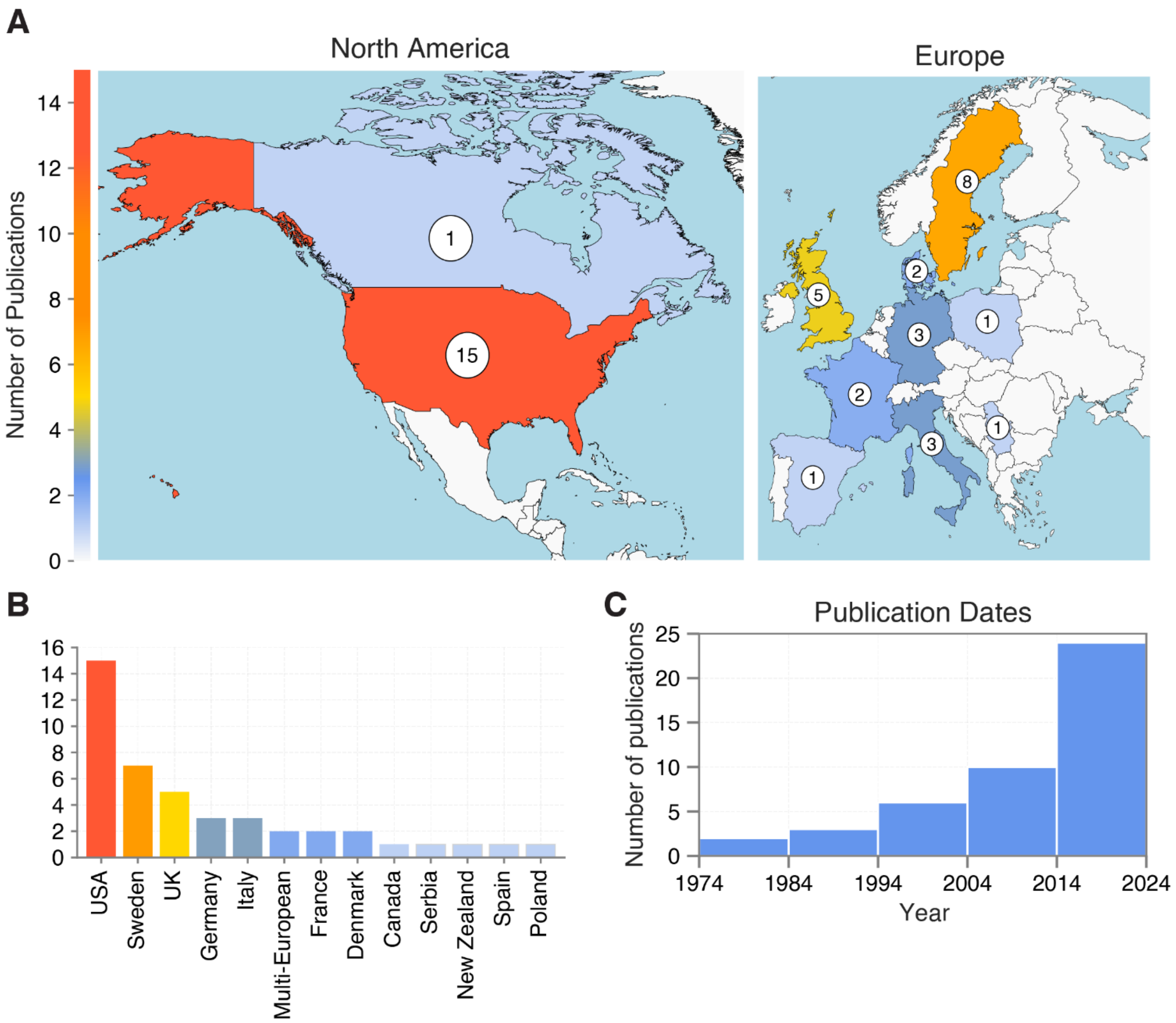

Supplementary Figure 3: Date and geographical distribution of studies. A. Geographical distribution of the selected studies, showing the number of studies conducted in different regions of North America and Europe. The colour gradient represents the number of publications, with the legend indicating the specific count. **B.** Distribution of publication dates for the studies included in this review (n=45). **C.** Distribution of publication dates for the selected studies.

Supplementary Figure 4

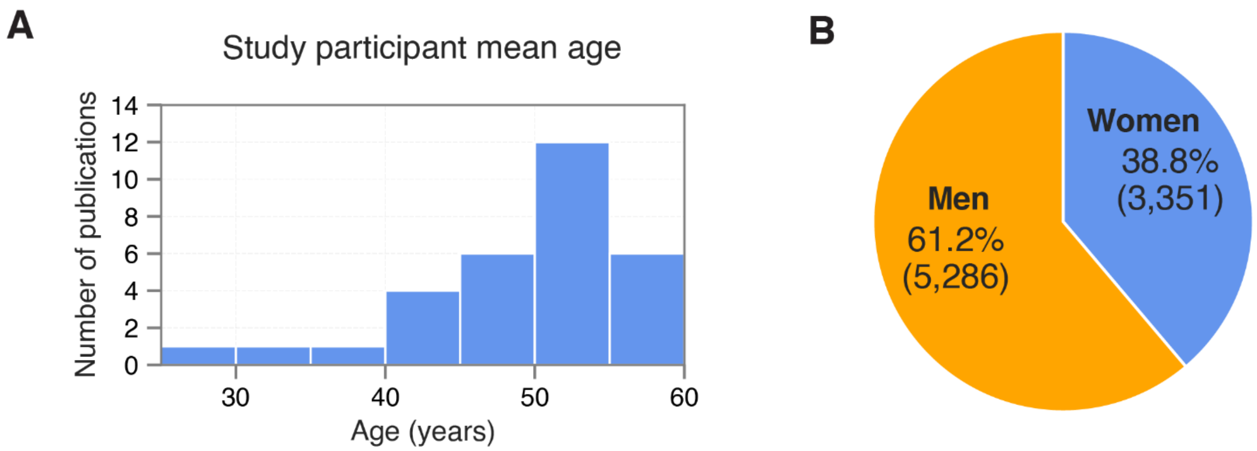

Supplementary Figure 4: Sex and age distribution of study participants. A. Mean study participant age, **B.** Sex distributions of the study participants.

Supplementary Figure 5:

The Newcastle-Ottawa Scale

The Newcastle-Ottawa Scale assesses non-randomised study quality on a 9-point scale focusing on selection, comparability, and outcome. Scoring thresholds are as follows: 0-3 indicates a high risk of bias, 4-5 a moderate risk, and 6-9 a low risk. These categories help systematically evaluate and interpret the methodological robustness of studies included in our meta-analysis.

	Questions	Scoring
Selection	Is sample representative of A1ATD PiZZ cohort? *(maximum 1 point)*	A: Fully representative **(1 point)**<br>B: Somewhat representative **(1 point)**<br>C: Not representative<br>D: No description
	Is control cohort drawn from the same community as the exposed cohort? *(maximum 1 point)*	A: Drawn from the same community as the exposed cohort **(1 point)**<br>B: Drawn from a different source<br>C: No description
	How was A1ATD PiZZ genotype ascertained? *(maximum 1 point)*	A: Clinically diagnosed cases by genotype/phenotype **(1 point)**<br>B: Existing registry data<br>C: Self-reported<br>D: No description
	Was the outcome of interest present at the start of the study? *(maximum 1 point)*	A: Yes **(1 point)**<br>B: No
Comparability	Were cohorts comparable on the basis of the design or analysis? *(maximum 2 points)*	A: The study controls for age and sex **(1 point)**<br>B: Study controls for additional factors (please list) **(1 point)**<br>C: Cohorts are not comparable; no controls described
Outcome	How was outcome of interest ascertained? *(maximum 1 point)*	A: Structured clinical assessment **(1 point)**<br>B: Clinical record **(1 point)**<br>C: Self-report<br>D: No description
	Was follow-up long enough for outcomes to occur? *(maximum 1 point)*	A: Yes **(1 point)**<br>B: No or not described
	Was follow-up cohort adequate? *(maximum 1 point)*	A: Complete follow up for all **(1 point)**<br>B: ≤20% follow up dropout **(1 point)**<br>C: ≥20% follow up dropout<br>D: No statement

Assessment summary

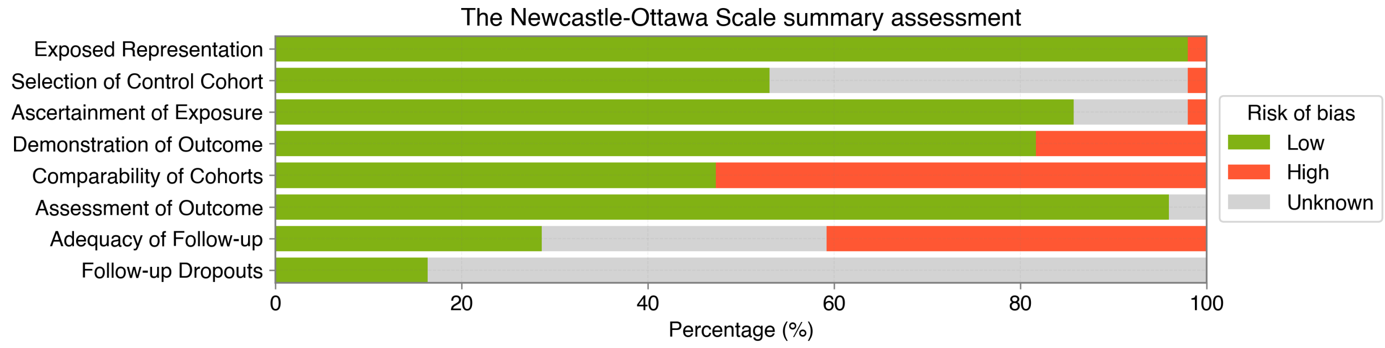

Bias scoring: Low (green) · High (red) · Unknown (grey)

Study	Selection: Exposed Representation	Selection: Selection of Control Cohort	Selection: Ascertainment of Exposure	Selection: Demonstration of Outcome	Comparability: Comparability of Cohorts	Outcome: Assessment of Outcome	Outcome: Adequacy of Follow-up	Outcome: Follow-up Dropouts	Score	Overall bias risk
Abboud (1979)	-	+	?	+	-	+	-	?	3	High
Beletic (2014)	+	+	+	+	-	+	-	?	5	Moderate
Bornhorst (2013)	+	?	+	+	-	+	-	?	4	Moderate
Bowlus (2005)	+	?	+	+	-	?	-	?	3	High
Brantly (1988)	+	?	+	+	-	+	-	?	4	Moderate
Carey (2013)	+	+	+	+	+	+	+	+	8	Low
Carleo (2016)	+	+	?	+	+	+	-	?	5	Moderate
Dawkins (2003)	+	?	?	-	-	+	+	+	4	Moderate
Dawwas (2012)	+	?	+	+	-	+	?	?	4	Moderate
Elzouki (1996)	+	+	+	+	+	+	?	?	6	Low
Eriksson (1987)	+	?	?	+	-	+	-	?	3	High
Felding (1980)	+	+	+	+	-	+	-	?	5	Moderate
Fromme (2022b)	+	+	+	+	+	+	?	?	6	Low
Fromme (2022a)	+	+	+	+	++	+	?	?	7	Low
Ghio (2013)	+	+	+	+	+	+	-	?	6	Low
Ghio (2013)	+	+	+	+	+	+	?	?	6	Low
Guillaud (2019)	+	?	+	+	-	+	?	?	4	Moderate
Hakim (2021)	+	+	+	+	+	+	-	?	6	Low
Hamesch (2017)	+	?	?	+	-	+	?	?	3	High
Hamesch (2019b)	+	?	+	+	-	+	?	?	4	Moderate
Hamesch (2019a)	+	+	+	+	++	+	?	?	7	Low
Hiller (2022)	+	+	+	+	+	+	+	+	8	Low
Hollander (2007)	+	+	+	+	+	+	-	?	6	Low
Holme (2010)	+	?	+	-	-	+	+	?	4	Moderate
Janus (1985)	+	+	+	-	-	+	+	?	5	Moderate
Malerba (2003)	+	+	+	+	+	+	-	?	6	Low
Montanello (2001)	+	+	+	+	-	+	-	?	5	Moderate
Morer (2016)	+	?	+	+	-	+	?	?	4	Moderate
Nunez (2021a)	+	+	+	+	++	+	?	?	7	Low
Nunez (2021b)	+	+	+	+	-	+	-	?	5	Moderate
Piitulainen (2005)	+	-	+	-	-	+	+	+	5	Moderate
Riis (2021)	+	+	+	-	+	+	+	?	6	Low
Sark (2022)	+	?	+	+	-	+	?	?	4	Moderate
Schneider (2020a)	+	+	+	+	++	+	?	?	7	Low
Schneider (2020c)	+	?	+	+	++	+	?	?	6	Low
Schneider (2020b)	+	+	+	+	++	+	?	?	7	Low
Shen (2022)	+	?	+	+	-	+	-	?	4	Moderate
Sitkauskiene (2008)	+	+	+	+	+	+	-	?	6	Low
Stoller (1994)	+	?	-	+	-	?	-	?	2	High
Stoller (2005)	+	?	+	-	-	+	+	+	5	Moderate
Suri (2023)	+	?	?	-	-	+	+	?	3	High
Tanash (2015)	+	+	+	-	-	+	+	+	6	Low
Tanash (2019)	+	?	+	-	-	+	+	?	4	Moderate
Teckman (2019)	+	?	+	+	-	+	+	+	6	Low
Vizzardi (2015)	+	?	+	+	+	+	-	?	5	Moderate
Wang (2019)	+	?	+	+	-	+	-	?	4	Moderate
Winther (2022)	+	+	+	+	+	+	-	?	6	Low
Wu (2023)	+	+	+	+	-	+	+	+	7	Low
von Schonfeld (1996)	+	?	+	+	-	+	+	?	5	Moderate

* - suffix letters in study year indicate multiple cohorts in a single study

Supplementary Figure 6

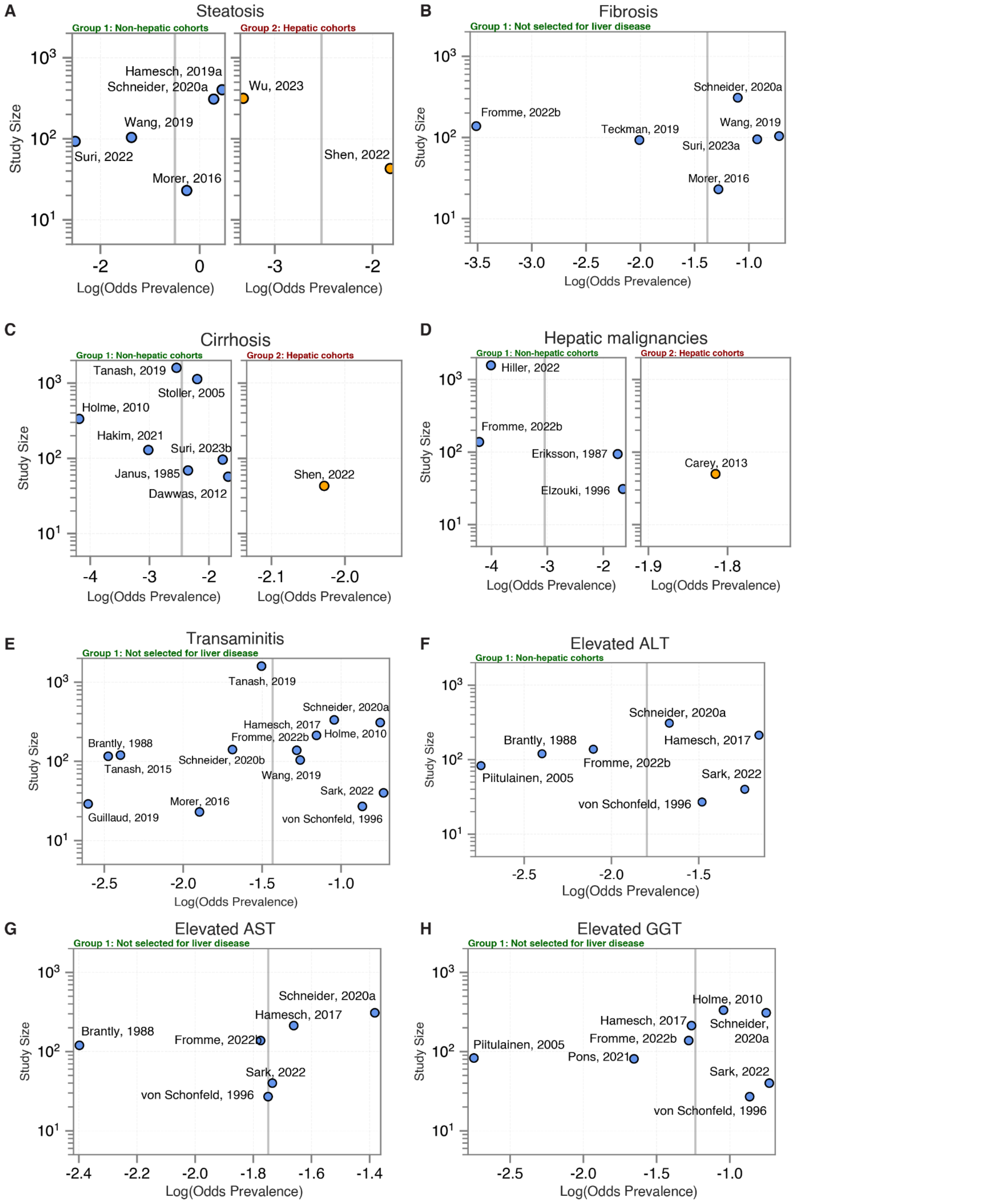

Supplementary Figure 6: Funnel plots of log odds of prevalence versus study size. Outlier studies were evaluated in-depth for relevance and potential sources of bias.

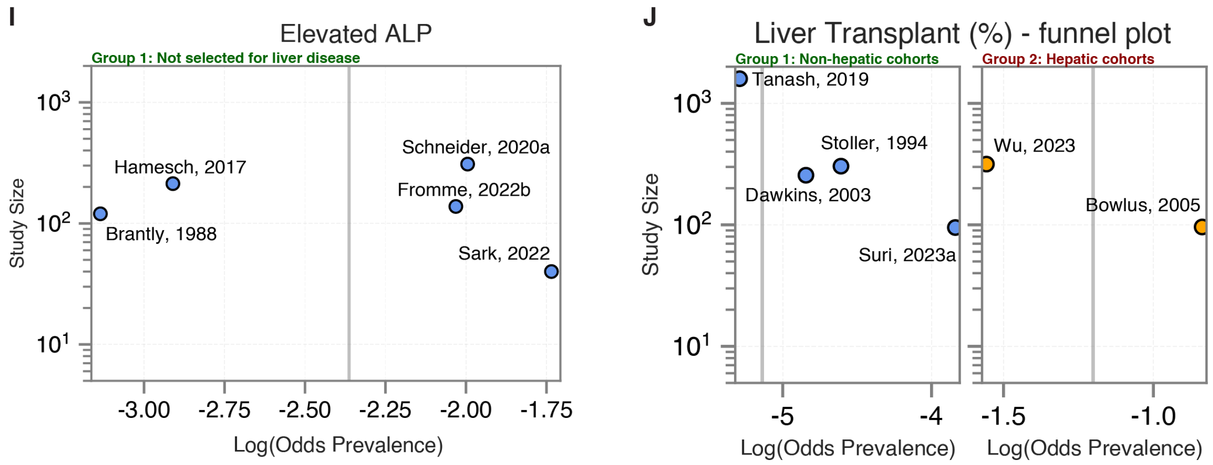

Supplementary Figure 6 continued: Funnel plots of log odds of prevalence versus study size. Outlier studies were evaluated in-depth for relevance and potential sources of bias.

Supplementary Table 1

First Author	Year	Country	Full title	DOI	PubMed ID	Study Design	ZZ cases (n)	Comparator (n)	Ethnicity (ZZ, %)	Recruitment	Prevalence statistics	Odds ratio and standardised mean difference statistics
Abboud	1979	Canada	Interrelationships between neutrophil elastase, serum alpha1-antitrypsin, lung function and chest radiography in patients with chronic airflow obstruction	10.1164/arrd.1979.120.1.31	313728	Cohort Study – Prospective	5	26	-	Non-hepatic recruitment	-	AAT SMD: -3.38 [95% CI: -4.70, -2.07]
Beletic	2014	Serbia	Is an integrative laboratory algorithm more effective in detecting alpha-1-antitrypsin deficiency in patients with premature chronic obstructive pulmonary disease than AAT concentration-based screening approach?	10.11613/BM.2014.032	24969923	Cohort Study – Retrospective	5	33	-	Non-hepatic recruitment	-	AAT SMD: -3.34 [95% CI: -4.57, -2.11]
Bornhorst	2013	USA	A1-antitrypsin phenotypes and associated serum protein concentrations in a large clinical population	10.1378/chest.12-0564	23632999	Cohort Study – Retrospective	814	58,087	-	Non-hepatic recruitment	-	AAT SMD: -3.88 [95% CI: -3.95, -3.81]
Bowlus	2005	USA	Factors associated with advanced liver disease in adults with alpha1-antitrypsin deficiency	10.1016/s1542-3565(05)00082-0	15822045	Cohort Study – Retrospective	96	23	-	Hepatic recruitment	Liver transplantation: 30.21%	-
Brantly	1988	USA	Clinical features and history of the destructive lung disease associated with alpha-1-antitrypsin deficiency of adults with pulmonary symptoms	10.1164/ajrccm/138.2.327	3264124	Cohort Study – Retrospective	120	-	99.2	Non-hepatic recruitment	Transaminitis: 8.33%	-
Carey	2013	USA	Outcomes for recipients of liver transplantation for alpha-1-antitrypsin deficiency–related cirrhosis	10.1002/lt.23744	24019185	Cohort Study – Retrospective	50	50	-	Hepatic recruitment	Liver cancer: 14.00%	-
Carleo	2016	Poland	Does urinary peptide content differ between COPD patients with and without inherited alpha-1 antitrypsin deficiency?	10.2147/COPD.S125240	28331304	Cohort Study – Retrospective	12	7	-	Non-hepatic recruitment	-	AAT SMD: -9.62 [95% CI: -13.18, -6.06]
Dawkins	2003	UK	Predictors of mortality in α1-antitrypsin deficiency	10.1136/thorax.58.12.1020	14645964	Cohort Study – Prospective	256	-	-	Non-hepatic recruitment	Liver transplantation: 0.78%	-
Dawwas	2012	UK	Prevalence and risk factors for liver involvement in individuals with PiZZ-related lung disease	10.1164/rccm.201204-0739oc	23262512	Cohort Study – Prospective	57	-	-	Non-hepatic recruitment	Cirrhosis: 15.79%	-

Elzouki	1996	Sweden	Risk of hepatobiliary disease in adults with severe α1-antitrypsin deficiency (PiZZ): is chronic viral hepatitis B or C an additional risk factor for cirrhosis and hepatocellular carcinoma?	10.1097/00042737-199610000-00010	8930564	Case Control Study	31	124	-	Non-hepatic recruitment	Cirrhosis: 41.94%<br>Liver cancer: 16.13%	Cirrhosis: cOR 10.47 [95% CI: 3.81, 28.78]<br>Liver cancer: cOR 5.77 [95% CI: 1.45, 22.97]
Eriksson	1987	Sweden	Alpha 1-antitrypsin deficiency and liver cirrhosis in adults: an analysis of 35 Swedish autopsied cases	Not available	3496734	Cohort Study – Retrospective	94	-	-	Non-hepatic recruitment	Liver cancer: 14.89%	-
Felding	1980	Sweden	Plasma prealbumin concentration in alpha-1 antitrypsin deficiency (PiZ)	10.1016/0026-0495(80)90146-8	6969840	Cohort Study – Retrospective	18	26	-	Non-hepatic recruitment	-	AAT SMD: -5.77 [95% CI: -7.17, -4.37]
Fromme	2022	Multi-European	Hepatobiliary phenotypes of adults with alpha-1 antitrypsin deficiency	10.1136/gutjnl-2020-323729	33632708	Cohort Study – Prospective	586	279	-	Non-hepatic recruitment	Fibrosis: 2.90%<br>Liver cancer: 1.45%<br>Transaminitis: 21.74%	Fibrosis: cOR 14.90 [95% CI: 5.50, 40.36]<br>Liver cancer: cOR 29.43 [95% CI: 7.24, 119.66]<br>Elevated ALT: cOR 1.78 [95% CI: 1.04, 3.05]<br>Elevated AST: cOR 3.68 [95% CI: 2.29, 5.92]<br>Elevated GGT: cOR 1.44 [95% CI: 0.96, 2.15]<br>Elevated ALP: cOR 1.06 [95% CI: 0.63, 1.79]
Ghio	2013	USA	Deficiency of α1-antitrypsin influences systemic iron homeostasis	10.2147/COPD.S37897	23378755	Cohort Study – Retrospective	30	30	83.3	Non-hepatic recruitment	-	AAT SMD: -23.77 [95% CI: -28.20, -19.34]
Guillaud	2019	France	Assessment of liver fibrosis by transient elastography (Fibroscan®) in patients with A1AT deficiency	10.1016/j.clinre.2018.08.016	30612958	Cohort Study – Prospective	29	-	-	Non-hepatic recruitment	Transaminitis: 6.90%	-
Hakim	2021	USA	Heterozygosity of the alpha 1-antitrypsin Pi*Z allele and risk of liver disease	10.1002/hep4.1718	34430780	Cohort Study – Retrospective	129	299,939	-	Non-hepatic recruitment	Cirrhosis: 4.65%	Cirrhosis: cOR 10.4 [95% CI: 4.58, 23.64]<br>aOR 11.83 [95% CI: 5.29, 26.48]
Hamesch	2019	Multi-European	Liver fibrosis and metabolic alterations in adults with alpha-1-antitrypsin deficiency caused by the Pi*ZZ mutation	10.1053/j.gastro.2019.05.013	31121167	Cohort Study – Retrospective	403	234	-	Non-hepatic recruitment	Steatosis: 61.04%	Steatosis: cOR 1.68 [95%CI: 1.21, 2.32]
Hamesch	2017	Germany	Multi-center study of liver disease in alpha1-antitrypsin deficiency: non-invasive evaluation of liver fibrosis in homozygous PiZZ patients	10.1016/S0168-8278(17)30637-2	Not available	Cohort Study – Prospective	213	-	-	Non-hepatic recruitment	Transaminitis: 23.94%	-
Hiller	2022	Sweden	Risk of cancer in individuals with severe alpha-1-antitrypsin deficiency (PiZZ) compared with the Swedish general population	10.1183/13993003.03200-2021	Not available	Cohort Study - Prospective	1,570	5,951	-	Non-hepatic recruitment	Liver cancer: 1.78%	Liver cancer: cOR 15.42 [95% CI: 6.72, 35.37]

Hollander	2007	Sweden	Plasma levels of alpha1-antichymotrypsin and secretory leukocyte proteinase inhibitor in healthy and chronic obstructive pulmonary disease (COPD) subjects with and without severe α1-antitrypsin deficiency	10.1186/1471-2466-7-1	17711594	Cohort Study - Retrospective	48	57	-	Non-hepatic recruitment	-	AAT SMD: -2.71 [95% CI: -3.24, -2.17]
Holme	2010	UK	Studies of gamma-glutamyl transferase in alpha-1 antitrypsin deficiency	10.3109/15412551003631733	20397813	Cohort Study – Prospective	334	-	-	Non-hepatic recruitment	Cirrhosis: 1.50% Transaminitis: 26.05%	-
Janus	1985	New Zealand	Smoking, lung function, and α1-antitrypsin deficiency	10.1016/S0140-6736(85)91916-6	2857224	Cohort Study – Prospective	69	-	-	Non-hepatic recruitment	Cirrhosis: 8.70%	-
Malerba	2003	Italy	Airway hyperresponsiveness in a large group of subjects with alpha1-antitrypsin deficiency: a cross-sectional controlled study	10.1046/j.1365-2796.2003.01083.x	12603503	Cohort Study – Retrospective	24	27	-	Non-hepatic recruitment	-	AAT SMD: -3.01 [95% CI: -3.83, -2.19]
Montanello	2001	Italy	Alpha-1-antitrypsin deficiency and nephropathy	10.1159/000046325	11744816	Cohort Study – Prospective	12	10	-	Non-hepatic recruitment	-	AAT SMD: -5.10 [95% CI: -6.96, -3.23]
Morer	2016	France	Liver involvement in patients with PiZZ-emphysema candidates for lung transplantation	10.1111/ajt.14152	27931086	Cohort Study – Retrospective	23	-	-	Non-hepatic recruitment	Steatosis: 43.48% Fibrosis: 21.74% Transaminitis: 13.04%	-
Nunez	2021	Spain	Association between circulating alpha-1 antitrypsin polymers and lung and liver disease	10.1186/s12931-021-01842-5	34526035	Cross-sectional	21	35	-	Non-hepatic recruitment	-	AAT SMD: -5.37 [95% CI: -6.54, -4.21]
Piitulainen	2005	Sweden	1-antitrypsin deficiency in 26-year-old subjects	10.1378/chest.128.4.2076	16236857	Cohort Study – Prospective	83	44	-	Non-hepatic recruitment	-	AAT SMD: -6.43 [95% CI: -7.31, -5.55]
Riis	2021	Denmark	α1-Antitrypsin Z allele and risk of venous thromboembolism in the general population	10.1111/jth.15556	34662507	Cohort Study – Prospective	55	101,365	100	Non-hepatic recruitment	-	AAT SMD: -14.58 [95% CI: -14.85, -14.31]
Sark	2022	Germany	The relationship between plasma alpha-1-antitrypsin polymers and lung or liver function in ZZ alpha-1-antitrypsin-deficient patients	10.3390/biom12030380	35327571	Cohort Study – Retrospective	40	-	-	Non-hepatic recruitment	Transaminitis: 32.50%	-

Schneider (a) (EU cohort)	2020	Multi-European	Liver phenotypes of European adults heterozygous or homozygous for Pi*Z variant of AAT (Pi*MZ vs Pi*ZZ genotype) and noncarriers	10.1053/j.gastro.2020.04.058	32376409	Cohort Study – Prospective	309	284	-	Non-hepatic recruitment	Steatosis: 56.96%<br>Fibrosis: 24.92%<br>Transaminitis: 32.04%	Steatosis: cOR 1.38 [95%CI: 1.0, 1.91]<br>Fibrosis: cOR 8.24 [95% CI: 4.28, 15.87]<br>AAT SMD: -4.19 [95% CI: -4.48, -3.90]<br>Elevated ALT: cOR 2.49 [95% CI: 1.44, 4.30]<br>Elevated AST: cOR 11.63 [95% CI: 4.94, 27.36]<br>Elevated GGT: cOR 3.85 [95% CI: 2.47, 5.99]<br>Elevated ALP: cOR 3.38 [95% CI: 1.69, 6.76]
Schneider (b) (UK biobank cohort)	2020	Multi-European	Liver phenotypes of European adults heterozygous or homozygous for Pi*Z variant of AAT (Pi*MZ vs Pi*ZZ genotype) and noncarriers	10.1053/j.gastro.2020.04.058	32376409	Cohort Study – Prospective	141	427,310	-	Hepatic recruitment	Transaminitis: 15.60%	-
Shen	2022	USA	Disease progression in patients with PI*ZZ alpha-1 antitrypsin deficiency	10.1097/MEG.0000000000002395	35802526	Cohort Study – Retrospective	43	-	-	Hepatic recruitment	Steatosis: 13.95%<br>Cirrhosis: 11.36%	-
Sitkauskiene	2008	UK	Screening for alpha1-antitrypsin deficiency in Lithuanian patients with COPD	10.1016/j.rmed.2008.07.003	18722101	Cohort Study – Retrospective	8	1,076	-	Non-hepatic recruitment	-	AAT SMD: -3.39 [95% CI: -4.10, -2.68]
Stoller	1994	USA	Physical and social impact of alpha1-antitrypsin deficiency: results of a survey	10.3949/ccjm.61.6.461	7828337	Cohort Study – Retrospective	304	12	100	Non-hepatic recruitment	Liver transplantation: 0.99%	-
Stoller	2005	USA	Mortality in individuals with severe deficiency of α1-antitrypsin	10.1378/chest.127.4.1196	15821195	Cohort Study – Retrospective	1129	-	-	Non-hepatic recruitment	Cirrhosis: 10.01%	-
Suri	2023	USA	Serum Z polymer levels and factors affecting increased liver fibrosis are associated with future severe liver disease outcomes in a prospective cohort of adults with alpha-1-antitrypsin deficiency	Not available	Not available	Cohort Study – Prospective	96	-	-	Non-hepatic recruitment	Cirrhosis: 14.58%<br>Liver transplantation: 2.11%	-
Suri	2022	USA	Hepatic steatosis, but not metabolic syndrome, is associated with increased hepatic fibrosis in adults with ZZ alpha-1-antitrypsin deficiency	10.1002/hep.32697	Not available	Cohort Study – Prospective	93	-	-	Non-hepatic recruitment	Steatosis: 7.53%	-

Suri	2023	USA	Increased liver fibrosis is the key clinical factor associated with increased future severe liver disease outcomes in a prospective cohort of adults with alpha-1-antitrypsin deficiency	10.1016/s0016-5085(23)03991-4	Not available	Cohort Study – Retrospective	95	-	-	Hepatic recruitment	Fibrosis: 28.42%	-
Tanash	2015	Sweden	The Swedish α1-antitrypsin screening study: health status and lung and liver function at age 34	10.1513/AnnalsATS.201410-452OC	25803183	Cohort Study – Prospective	116	229	-	Non-hepatic recruitment	Transaminitis: 7.76%	-
Tanash	2019	Sweden	Liver disease in adults with severe alpha-1-antitrypsin deficiency	10.1007/s00535-019-01548-y	30680526	Cohort Study – Retrospective	1595	-	-	Non-hepatic recruitment	Cirrhosis: 7.27%<br>Transaminitis: 18.18%<br>Liver transplantation: 0.50%	-
Teckman	2019	USA	Clustering at the extremes of mild and severe fibrosis is seen on liver biopsy in preliminary analysis of a multi-center, prospective adult cohort of alpha-1-antitrypsin deficiency	Not available	Not available	Cohort Study – Prospective	93	-	-	Non-hepatic recruitment	Fibrosis: 11.83%	-
Vizzardi	2015	Italy	Echocardiographic evaluation in subjects with α1-antitrypsin deficiency	10.1111/eci.12492	26257247	Cohort Study – Prospective	33	33	-	Non-hepatic recruitment	-	AAT SMD: -13.10 [95% CI: -15.45, -10.74]
von Schonfeld	1996	Germany	Liver function in patients with pulmonary emphysema due to severe alpha-1-antitrypsin deficiency (PiZZ)	10.1159/000201331	8739089	Cohort Study – Prospective	27	-	-	Non-hepatic recruitment	Transaminitis: 29.63%	-
Wang	2019	USA	Alpha-1 antitrypsin deficiency liver disease: mutational homogeneity modulated by epigenetic heterogeneity with links to obesity	10.1002/hep.30526	30681738	Cohort Study – Prospective	104	-	100	Non-hepatic recruitment	Steatosis: 20.19%<br>Fibrosis: 32.69%<br>Transaminitis: 22.12%	-
Winther	2022	Denmark	Severe α1-antitrypsin deficiency associated with lower blood pressure and reduced risk of ischemic heart disease: a cohort study of 91,540 individuals and a meta-analysis	10.1186/s12931-022-01973-3	35264159	Cohort Study – Retrospective	185	91,148	-	Non-hepatic recruitment	-	AAT SMD: -4.71 [95% CI: -4.85, -4.56]
Wu	2023	USA	Liver disease progression in patients with alpha-1 antitrypsin deficiency and protease inhibitor ZZ genotype with or without lung disease	10.1111/apt.17715	37718576	Cohort Study - Retrospective	316	-	96.8	Hepatic recruitment	Steatosis: 3.48%<br>Liver transplantation: 17.41%	-
